# Cystic colon duplication causing intussusception in a 25-year-old man: report of a case and review of the literature

**DOI:** 10.1186/1471-2482-10-19

**Published:** 2010-06-23

**Authors:** Carolin Reiser-Erkan, Mert Erkan, Erika Ulbrich, Jörg Nährig, Jörg Kleeff

**Affiliations:** 1 Department of General Surgery, Klinikum rechts der Isar, Technische Universität München, Ismaninger Strasse 22, 81675 Munich, Germany; 2 Department of Radiology, Klinikum rechts der Isar, Technische Universität München, Ismaninger Strasse 22, 81675 Munich, Germany; 3 Department of Pathology, Klinikum rechts der Isar, Technische Universität München, Ismaninger Strasse 22, 81675 Munich, Germany

## Abstract

**Background:**

Colonic intussusception is a rare congenital abnormality, mostly manifesting before the age of two with abdominal pain and acute intestinal obstruction with or without bleeding. In adults it may occur idiopathically or due to an intraluminal tumor mass.

**Case presentation:**

A 25-year-old man presented with an acute abdomen and severe crampy abdominal pain. The clinical picture mimicked acute appendicitis. Transabdominal ultrasound examination revealed a 5 cm circular mass in the right upper abdomen. The ensuing computed tomography suggested an intussusception in the ascending colon. Intraoperatively, no full thickness invagination was detected. Due to a hard, intraluminal tumor a standard right hemicolectomy with ileotransversostomy was performed. The histopathological analysis revealed a cystic colon duplication leading to mucosal invagination and obstruction.

**Conclusions:**

In adults, colon intussusception is a rare event causing approximately 1% of all acute intestinal obstructions. Unlike its preferentially nonsurgical management in children, a bowel intussusception in adults should be operated because an organic, often malignant lesion is present in most cases.

## Background

Colon intussusception and gastrointestinal duplications are diseases of young children occurring and/or becoming symptomatic usually within the first two years of life. The most typical site for colon intussusception is the ileo-ceacal region; similarly, gastrointestinal duplications most commonly affect the ileum. Surgery in case of intussusception is indicated, if bowel segments are at risk to become necrotic, with impending perforation and subsequent peritonitis [1]. Gastrointestinal duplications can manifest with signs of acute abdomen or acute bleeding, necessitating emergency surgery [2].

## Case presentation

**Figure 1:**
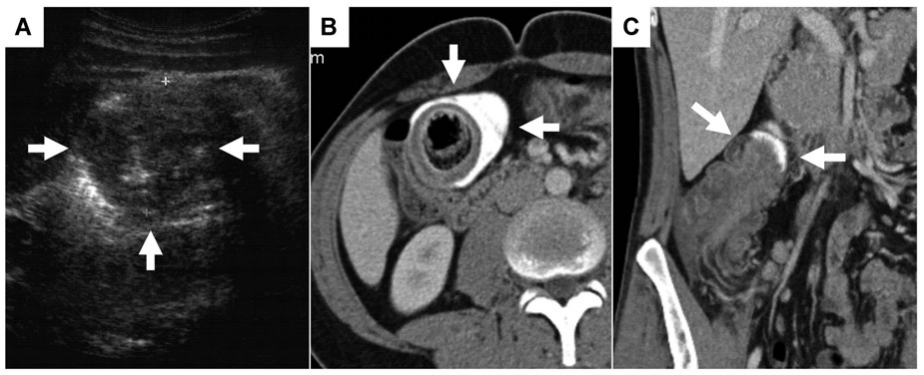
**Pre-operative imaging of the abdomen by ultrasound and computertomography**. A) Transabdominal 5MHz Ultrasound examination revealing a 47 mm diameter round mass in the right upper abdominal quadrant (arrows). The computed tomography of the abdomen in an axial plane (B) with coronal reconstruction (C) showing the cystic duplication with mucosal invagination in the ascending colon. Notice the rectal contrast filling that stops at the lesion (arrows).

A 25-year-old man presented with an acute abdomen with abdominal defence and rebound in the right lower quadrant and with severe crampy abdominal pain. The patient reported of blood mixed stool within the last four days. There was no history or family history of inflammatory bowel disease. A transabdominal ultrasound examination revealed an unclear circular mass with 5 cm diameter in the right upper quadrant. The subsequent CT-scan showed an invagination of the ascending colon with rectal contrast enema passing no further (Figure 1); there was no critical extent of ceacal dilation evident on the CT-scan. No other intraabdominal pathology was detected.

**Figure 2:**
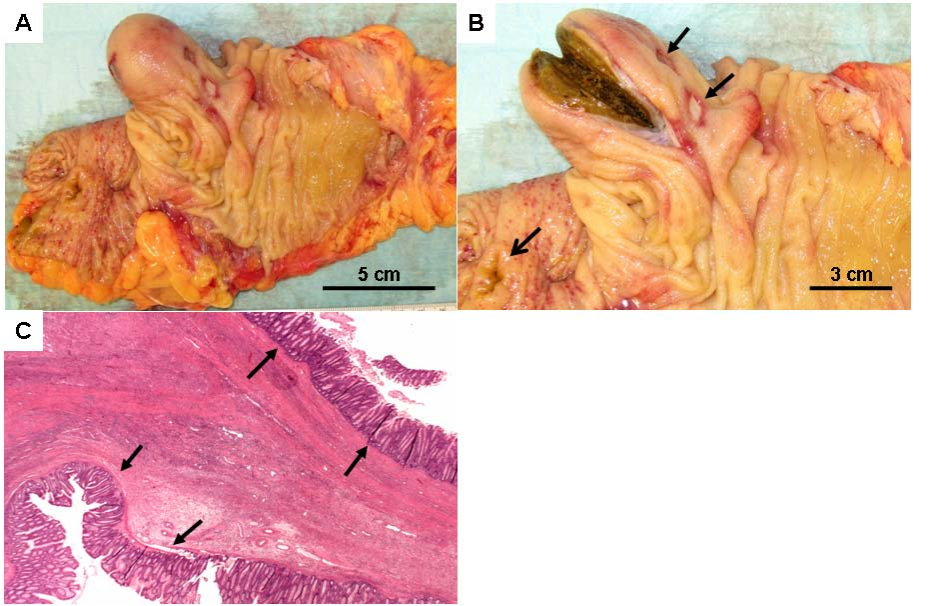
**Macroscopical and histopathological findings of the specimen**. A) The right hemicolon opened with the cystic duplication. B) The stool-filled cystic lesion is cut open. Notice the mucosal ulcerations (closed arrows). The open arrow depicts the ileo-ceacal area. C) Histopathological demonstration of the duplication cyst.

Intraoperatively, there was a firm intraluminal mass palpable in the ascending colon. A standard non-oncological right-hemicolectomy was performed. Intraoperative opening of the specimen revealed a circular, mucosa covered intraluminal mass acting as the leading point that caused partial mucosal invagination and obstruction. The stool filled saccular lesion had ulcerous areas visible on the mucosal surface (Figure 2A, B). Since there was no macroscopic evidence of malignancy, no further radical lymph-node dissection was made and the bowel continuity was re-established by an ileotransversostomy. The postoperative course was uneventful. Histopathological examination revealed a cystic colonic duplication (Figure 2C).

## Conclusions

Colon intussusception mostly occurs in children up to two years of age (boys to girls ratio: 3 to 1) due to intermittent changes in bowel motility. Although it may happen in any segment of the bowel, the most typical site is the ileo-ceacal region. Early symptoms of bowel obstruction include nausea, bilious vomiting, and intermittent moderate to severe cramping abdominal pain. Later symptoms include trans-rectal bleeding, or stool mixed with mucus and blood. Palpation of the abdomen may show a mass in the region of the punctum maximum of pain. Due to intussusception, segments may become necrotic, which can lead to bowel perforation and peritonitis [1]. Diagnosis is typically made by careful history and physical examination. A definite diagnosis often requires confirmation by imaging modalities. To evaluate intestinal obstruction or exclude free intraperitoneal air an x-ray examination is the initial examination. In children ultrasound is considered the diagnostic imaging modality of choice due to its high accuracy (target-like mass) and lack of irradiation. In adults a CT-scan is mostly performed [3]. Emergency treatment is necessary to prevent bowel ischemia and necrosis, which then requires surgical resection. An air enema may be used to "relocate" the bowel only in children.

Gastrointestinal duplications are uncommon congenital abnormalities that manifest before the age of 2 years in 80% of cases. Ileal duplication is the most common one, while colonic duplications, either cystic (>80%) or tubular, occur in 10%-15% of cases and remain asymptomatic and undiagnosed in most instances. Symptomatic colonic duplication is a rarity in adults. The most common clinical symptoms are abdominal pain and intestinal obstruction. Sometimes, duplications present with signs of acute abdomen or acute bleeding, necessitating emergency surgery [2].

Both events are unusual especially in adults, the combination of both is even more uncommon [4,5]. Adults with intussusception (as well as with enteric duplications) are presenting with various non-specific symptoms of bowel obstruction, like abdominal pain, nausea and constipation [1]. These symptoms can be acute, subacute or chronic. CT scanning is the most sensitive diagnostic modality. Intussusception displays a pathognomonic appearance of a complex soft tissue mass, consisting of the outer intussuscepiens and the central intussusceptum. When the CT beam is parallel to the intussusception's longitudinal axis, it appears as a "sausage-shaped" mass; when the beam is perpendicular to its axis it appears as a "target" mass [3]. Because of the mostly non-specific symptoms of both entities, it is often difficult to diagnose an intussusception/colonic duplication before operation. Hanan et al. recently reported about 16 cases, only in 8 (50%) of them the right diagnosis was made preoperatively [1,6].

In the reported case we chose to operate the patient because of the clinical presentation with an acute abdomen. The definitive diagnosis of colonic intussusception and duplication could not be made preoperatively. The acute symptoms of (partial) obstruction were lasting for already 4 days, so that the risk of perforation was high. Unlike in children with a high incidence of idiopathic intussusception, in adults intussusception is often the first symptom of a malignancy. Thus, in up to 50% of colonic intussusceptions in adults, the colonic lesions are malignant (adenocarcinoma), so the definitive treatment should be oncological surgery with the resection of the affected bowel segment [7,8]. A non-oncologic right-sided hemicolectomy was performed, because macroscopically there was no evidence for malignancy. Arguably, this was a macroscopic assessment of the attending surgeon that exposed the patient to a potentially not curative resection. Clearly, if the dignity of the lesion is in doubt, an oncological operation has to be carried out [9].

In conclusion, bowel intussusceptions and gastrointestinal duplications are rare but have to be kept in mind in the differential diagnosis of an acute abdomen in adults. As in every abdominal mass lesion, an underlying malignancy has to be considered. Therefore an oncological operation should be planned, especially for right-sided lesions, since in this location oncological resections can be performed without impairment of postoperative bowel functions or increased morbidity.

## Competing interests

There was no financial competing interest for any of the authors, as well as there was no non-financial competing interest (political, personal, religious, ideological, academic, intellectual, commercial or any other).

## Authors' contributions

CR-E, JK designed and initiated the study. CR-E, ME, JK performed a literature search. EU performed the radiological analysis. JN performed the pathological analysis. CR-E, ME, JK drafted and wrote the manuscript; EU, JN critically revised the manuscript. All authors read and approved the final version of the manuscript and agree with the manuscript's results and conclusions.

## Consent

Written consent was obtained from the patient for publication of this case report and any accompanying images. A copy of the written consent is available for review by the Editor-in-Chief of this journal.

## Pre-publication history

The pre-publication history for this paper can be accessed here:

http://www.biomedcentral.com/1471-2482/10/19/prepub
